# Al-Ti-Containing Lightweight High-Entropy Alloys for Intermediate Temperature Applications

**DOI:** 10.3390/e20050355

**Published:** 2018-05-09

**Authors:** Minju Kang, Ka Ram Lim, Jong Woo Won, Kwang Seok Lee, Young Sang Na

**Affiliations:** Korea Institute of Materials Science, 797 Changwondae-ro, Seongsan-gu, Changwon, Gyeongnam 642-831, Korea

**Keywords:** high-entropy alloys, alloys design, lightweight alloys

## Abstract

In this study, new high-entropy alloys (HEAs), which contain lightweight elements, namely Al and Ti, have been designed for intermediate temperature applications. Cr, Mo, and V were selected as the elements for the Al-Ti-containing HEAs by elemental screening using their binary phase diagrams. AlCrMoTi and AlCrMoTiV HEAs are confirmed as solid solutions with minor ordered B2 phases and have superb specific hardness when compared to that of commercial alloys. The present work demonstrates the desirable possibility for substitution of traditional materials that are applied at intermediate temperature to Al-Ti-containing lightweight HEAs.

## 1. Introduction

Recently, high-entropy alloys (HEAs) have attracted considerable attention because of their extraordinary properties [1,2,3,4,5,6,7,8,9,10], and numerous HEAs have been reported with various compositions [1,2,3,4,5,6,7,8,9,10]. Three major HEAs are CoCrFeMnNi alloy which has potential applications in cryogenic environments [5,6], refractory VNbMoTaW alloy for high-temperature structural applications [7,8], and AlCoCrFeNi alloy which maintains high strength up to intermediate temperatures [9,10]. AlCoCrFeNi HEA is relatively lightweight and has excellent specific strength around intermediate temperatures. It is a possible alternative for Ti alloys or wrought superalloys such as Inconel 718 [9,10]. Additional weight reduction can improve the competitiveness of the lightweight HEAs; therefore, we attempted to develop new HEAs that contain lightweight elements, namely Al and Ti. 

Most HEAs are developed by a trial-and-error approach based on the effects such as mixing enthalpy and valence electron concentration (VEC) [11,12]. This conventional method is not effective when developing HEAs with a new combination of elements among numerous possibilities. The CALPHAD (Computer Coupling of Phase Diagrams and Thermochemistry) approach may be the best way for designing new HEAs because of its capability in predicting the phase stability [13,14,15,16]. However, the application of CALPHAD to the design of new HEAs is challenging because of the lack of a reliable thermodynamic database to cover the entire composition range [13,14,15]. F. Zhang et al. [17] reported a new approach to design new multi-component FCC HEAs by binary phase diagrams. The FCC single phase formation in the CoCrFeMnNi HEA was predicted using this approach [17]. This method is effective at finding “matching elements” that form a single solid solution and is suitable for designing novel HEAs.

In this work, we sought to design lightweight HEAs, which contain lightweight elements, namely Al and Ti, by using binary phase diagrams. Seven HEAs containing Al and Ti were designed and their mechanical properties were compared with those of commercial alloys. 

## 2. Materials and Methods 

The selection of candidate elements was conducted based on their binary phase diagrams. The details regarding the design of the Al-Ti-containing lightweight HEAs are described in Section 3.1. 

The aforementioned Al-Ti-containing lightweight HEAs were fabricated by vacuum plasma arc melting (PAM) with high-purity elements. The HEA button ingots were re-melted 4 to 5 times in a melting furnace for the homogenization. The HEA plates were fabricated by vacuum induction melting using a graphite mold. The microstructures of the HEAs were analyzed by optical microscopy (OM), FE-SEM (model: SU-6600, HITACHI), and transmission electron microscopy (TEM, model: Tecnai F20, FEI). The TEM samples were prepared using focused ion beam (FIB). The crystal structure of the material was examined by X-ray diffraction (XRD) measurements on the as-casted material using a MXP21VAHF diffractometer with a CuKα radiation source (model: D/Max-2500VL/PC, RIGAKU). The Vickers hardness tests were carried out using a conventional indenter with a load of 2.94 N for 15 s. A minimum 10 tests were carried out on specimen.

## 3. Results and Discussion

### 3.1. Selection of Candidate Elements Based on Their Binary Phase Diagrams

**Figure 1:**
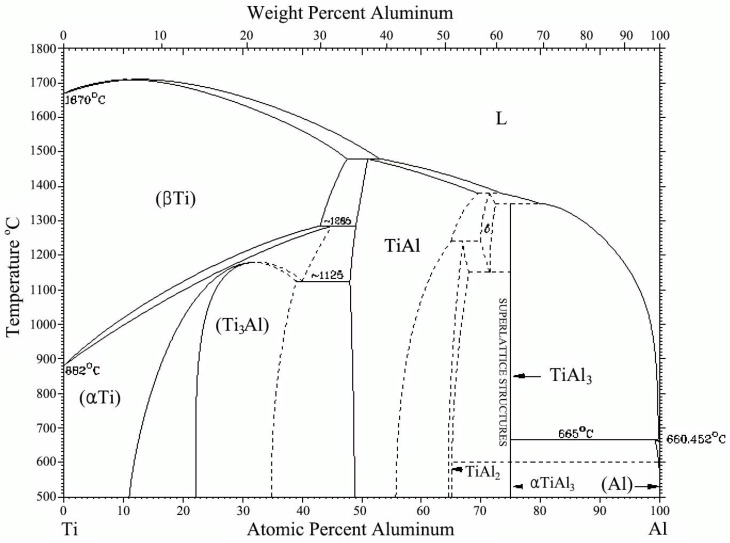
Al-Ti phase diagram [18]. It has solid solution region within a certain temperature and composition range that suggest the possibility of the formation of single solid solution.

To design lightweight HEAs, we selected Al and Ti as the basic elements owing to their low density. The key idea of designing new HEAs using their binary phase diagrams is finding elements that mix. Therefore, the solubility of Al and Ti should be investigated first. Figure 1 shows the Al-Ti phase diagram [18]. It is very complex, and numerous intermediate ordered phases exist. However, β-Ti (BCC phase) and α-Ti (HCP phase) appear in the Ti-rich region as a solid solution. These solid solution regions suggest the possibility of the formation of single solid solution. Therefore, the lightweight elements, Al and Ti, could be the basic elements for lightweight HEAs. 

The design of the Al-Ti-containing HEAs consisted of three steps. In the first step, the candidate elements were selected from various Ti-X binary phase diagrams wherein “X” represents elements that form homogeneous solid solution with Ti within a certain temperature range [18]. In the second step, a second series of candidate elements were selected from various Y-Al binary phase diagrams, wherein “Y” represents elements that show adequate solubility in Al within a certain composition and temperature range. The final step was the selection of the final candidate elements from the X-Y binary phase diagrams.

**Figure 2:**
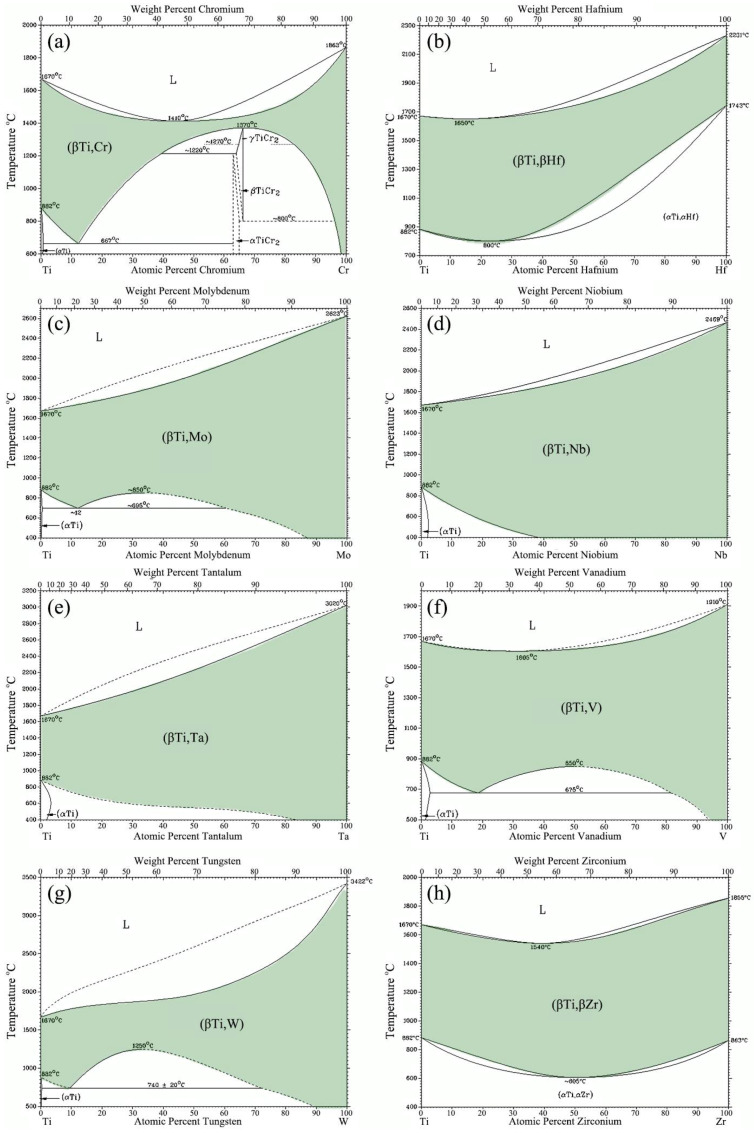
Ti-X phase diagrams where “X” is (**a**) Cr; (**b**) Hf; (**c**) Mo; (**d**) Nb; (**e**) Ta; (**f**) V; (**g**) W; and (**h**) Zr [18].

From the Ti-X binary phase diagrams, we found 8 candidate elements, Cr, Hf, Mo, Nb, Ta, V, W, and Zr, in which each had a solid solution region in a certain range [18]. Figure 2 shows the Ti-X (X = Cr, Hf, Mo, Nb, Ta, V, W, or Zr) binary phase diagrams. All elements had a large area of solid solution within a certain temperature range, and all the homogeneous solid solution phases had BCC crystalline structure, indicated in green (Figure 2). 

The next step consisted of finding a second series of candidate elements. Their binary phase diagrams are shown in the supplementary data (Figure S1). 6 candidate elements, Cr, Hf, Mo, Nb, V, and Zr, were chosen from the Y-Al binary phase diagrams. These elements are not form solid solution in all composition ranges, although the solid solutions contain Y’s own crystalline structure (BCC) at a certain temperature and composition range. Among the 6 candidates, Nb and Zr were removed because they form solid solution in very restricted range. 

From the first and second steps, Cr, Hf, Mo, and V were selected. To select the final candidate elements, the binary phase diagrams between these elements were investigated. Hf-Cr, Hf-Mo, and Hf-V showed complex phase diagrams, which could have possibly formed some intermediate ordered phases (supplementary data, Figure S2). Therefore, Cr, Mo, and V were selected as the elements for the Al-Ti-containing HEAs.

### 3.2. Microstructure of the Al-Ti-Containing HEAs

**Figure 3:**
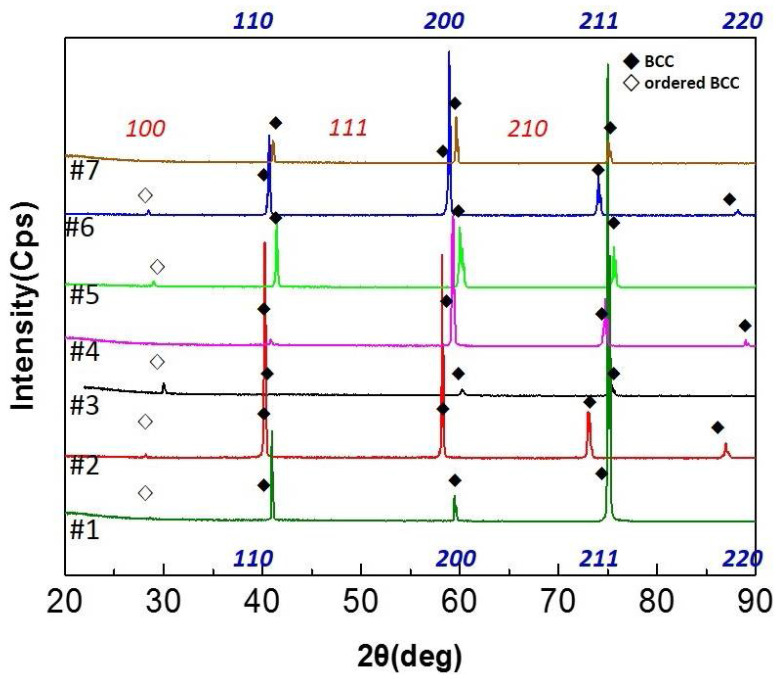
The XRD patterns of Al-Ti-containing HEAs.

Ternary, quaternary, and quinary HEAs were designed by adding the selected elements to Al-Ti. Seven HEAs, numbered #1 to #7, were fabricated and their compositions are detailed in Table 1. The XRD profiles of the Al-Ti-containing HEAs are shown in Figure 3. The AlCrMoTi (#4) and AlCrMoTiV (#7) had a single BCC structure, and ordered BCC peaks appeared for the other HEAs. 

**Figure 4:**
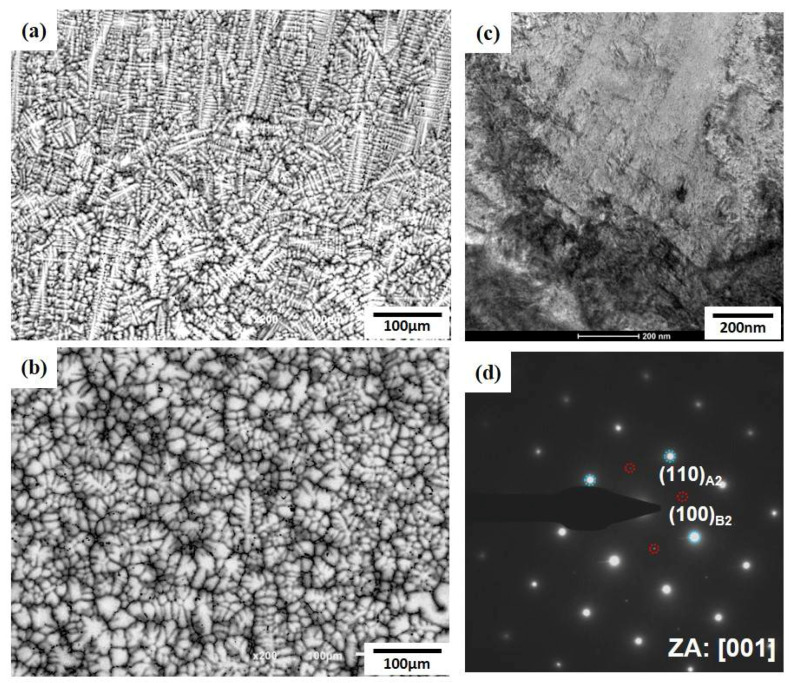
Microstructures of (**a**) AlCrMoTi and (**b**) AlCrMoTiV. Both have dendritic morphology. (**c**) bright field image and (**d**) diffraction pattern of AlCrMoTiV.

The microstructures of AlCrMoTi (#4) and AlCrMoTiV (#7) which had a single BCC structure, are shown in Figure 4a,b. Both showed a dendritic morphology, and the dendritic growth was suppressed by the addition of V. The TEM analysis was performed for AlCrMoTiV (#7), and the results are shown in Figure 4c,d. Figure 4c is bright field image of AlCrMoTiV (#7) and Figure 4d is the diffraction pattern taken from [001]_B2/BCC_ zone axis of Figure 4c. Although the XRD results demonstrate that it is a single BCC structure, diffraction pattern reveals (100) superlattice reflections marked with red-dotted circle that indicate an ordered B2 phase. Nano-scale B2 phases were observed; however, it was not detected by the XRD owing to their small fraction and size. Because AlCrMoTiV (#7) contained minor ordered precipitates, it could be defined as an ordered solid solution [19].

One of the physical properties of HEAs is the high-entropy effect [20]. The solid solution is stabilized because of the high configurational entropy [20,21], and this effect increases with an increased number of elements [20]. This trend can be observed in our work. As the number of elements increased from ternary to quinary, the phases progressively became simpler. The high-entropy effect is dominant at high temperatures according to G = H − TS, where G is Gibbs free energy, H is enthalpy, T is temperature, and S is entropy [20,21]. Thus, this HEA can exist as a solid solution at elevated temperatures, even though a small fraction of the B2 phase existed in an as-cast state at room temperature. 

### 3.3. Application of Empirical Parameters 

The empirical parameters for the HEAs originated from the classic Hume-Rothery rules [22,23]. Guo et al. [24] reported that −22 ≤ ΔH_mix_ ≤ 7 kJ/mol, and δ ≤ 8.5% are required for the sole simple phases (i.e., FCC, BCC, and their mixtures, including both ordered/disordered cases). Zhang et al. [19] proposed −15 ≤ ΔH_mix_ ≤ 5 kJ/mol and δ ≤ 6.5%, whereas Yang et al. [12] proposed δ ≤ 6.6%. In the case of VEC, 8 < VEC and VEC < 6.87 were suggested for the single FCC and BCC structures, respectively [20]. These parameters were statistically determined; therefore, there are differences and exceptions depending on the work [12,19,22,25].

**Figure 5:**
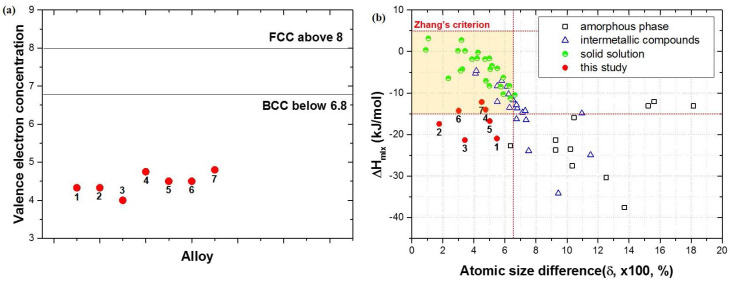
(**a**) VEC and (**b**) δ − ΔH_mix_ plot of Al-Ti-containing HEAs. Yellow colored regions highlight Zhang’s criterion [19] for single phase to form.

The empirical parameters, valence electron concentration (VEC), atomic size difference (δ), and enthalpy of mixing (ΔH_mix_) of the designed HEAs were calculated and are shown in Table 1 and Figure 5 [22]. The VEC and δ values satisfied the existing criteria. However, the ΔH_mix_ values were positioned relatively below the alloys which form solid solutions (Figure 5b). All the HEAs satisfy Guo’s criterion although only AlCrMoTi (#4), AlMoTiV (#6), and AlCrMoTiV (#7) satisfy Zhang’s criterion. The microstructural analysis demonstrates that AlCrMoTi (#4) and AlCrMoTiV (#7) are ordered solid solutions; the empirical parameters of HEAs in present work agree with previous research and support the idea that using the binary phase diagrams can be a solution to screen the proper candidate elements for the design of novel HEAs.

### 3.4. Specific Hardness of the Al-Ti-Containing HEAs 

**Figure 6:**
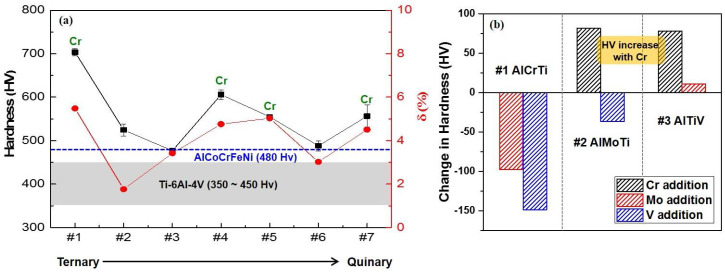
(**a**) Relationship between the hardness and atomic size difference; (**b**) hardness variation with the Cr, Mo, and V addition to ternary HEAs. The hardness increases remarkably with the Cr addition.

It has been reported that HEAs have a severe lattice distortion owing to the atomic size differences of elements, which induces solid solution strengthening [25]. Figure 6 shows the relationship between the hardness and δ. The hardness varied across the δ values. Moreover, Figure 6b clearly shows the hardness variation with the addition of Cr, Mo, and V. The hardness increased remarkably with the addition of Cr. The atomic radius of Cr is 1.25 Å which is quite different than the others [24]; this causes further severe lattice distortion. 

The hardness of the AlCrMoTi (#4) and AlCrMoTiV (#7) solid solutions were compared with that of other HEAs and commercial alloys and is summarized in Table 2 [26,27,28,29,30,31]. The hardness of the AlCrMoTi (#4) and AlCrMoTiV (#7) were 606 ± 11 and 556 ± 25 HV, respectively. These values are 17% and 7% higher than that of the AlCoCrFeNi HEA, which is representative of HEAs for intermediate temperature applications [9]. The theoretical densities are also lower than that of the AlCoCrFeNi HEA, so the specific hardnesses were 30% and 19% higher than that of the AlCoCrFeNi HEA. Furthermore, the specific hardness of the HEAs in the present work are considerably higher than that of competitive conventional alloy systems. The AlCrMoTi (#4) showed a 29% improved specific hardness when compared with that of Ti-6Al-4V alloy, which is an alloy that is commonly used for intermediate temperature applications. It is expected that a great improvement in the hardness can accomplish the weight lightening through the gage reduction of machineries despite their higher density when compared to that of the Ti-6Al-4V alloy. Further research on the fine-tuning of the elemental composition for lighter and cheaper alloys as well as a detailed microstructural and high-temperature property analysis could open a new path toward lightweight HEAs for intermediate temperature applications.

## 4. Conclusions

In summary, HEAs which contained Al and Ti were designed based on their binary phase diagrams. This approach is powerful for screening candidate elements for novel HEAs. The candidate elements that were selected formed a solid solution within a certain temperature and composition range. The high-entropy effect is enhanced with an increased number of elements; therefore, the AlCrMoTi and AlCrMoTiV HEAs are verified to be solid solutions with a minor ordered B2 phase. These HEAs have a superb specific hardness when compared to that of Ti-6Al-4V and Inconel 718 alloys and show promise as future substitutes for Ti alloys for intermediate temperature structural applications. Furthermore, fine-tuning of the elemental composition of HEAs can lead to the development of novel light-weight HEAs.

## Figures and Tables

**Table 1:** Valence electron concentration (VEC), atomic size difference (δ), and enthalpy of mixing (ΔH_mix_) of designed HEAs.

Alloys		VEC	δ (%)	ΔH_mix_ (kJ/mol)
#1	AlCrTi	4.33	5.49	−20.96
#2	AlMoTi	4.33	1.77	−17.44
#3	AlTiV	4.00	3.42	−21.33
#4	AlCrMoTi	4.75	4.76	−14.00
#5	AlCrTiV	4.50	5.03	−16.75
#6	AlMoTiV	4.50	3.02	−14.25
#7	AlCrMoTiV	4.80	4.51	−12.16

**Table 2:** Comparison of specific hardness of HEAs with other alloys.

Alloys	Hardness (HV)	Theoretical Density (g/cm^3^)	Specific Hardness (HV/g/cm^3^)
AlCrMoTi (#4)	606	6.01	100.83
AlCrMoTiV (#7)	556	6.00	92.67
AlCoCrFeNi [26]	520	6.70	77.61
AlCoCrCuFeNi [27]	400	7.02	56.98
Al_0.5_CoCrCuFeNiV [28]	640	7.27	88.03
Ti-6Al-4V [29]	346	4.43	78.10
Ti-6242 [30]	339	4.54	74.67
Inconel 718 [31]	355	8.18	43.40

## Supplementary Materials

The following are available online at http://www.mdpi.com/1099-4300/20/5/355/s1, Figure S1: Y-Al phase diagrams where “Y” is (a) Cr, (b) Hf, (c) Mo, (d) Nb, (e) V, and (f) Zr [18]. Figure S2: (a) Hf-Cr, (b) Cr-Mo, (c) Cr-V, (d) Hf-Mo, (e) Hf-V, and (f) Mo-V binary phase diagrams [18]. Cr, Mo, and V were selected which have simple phase diagrams.
