# Supplementary material for: Al-Ti-Containing Lightweight High-Entropy Alloys for Intermediate Temperature Applications
Source: Entropy (Basel). 2018 May 9;20(5):355. doi: 10.3390/e20050355 (PMC7512874; doi:10.3390/e20050355)
Supplement: Supplementary file 1 [file entropy-20-00355-s001.pdf]

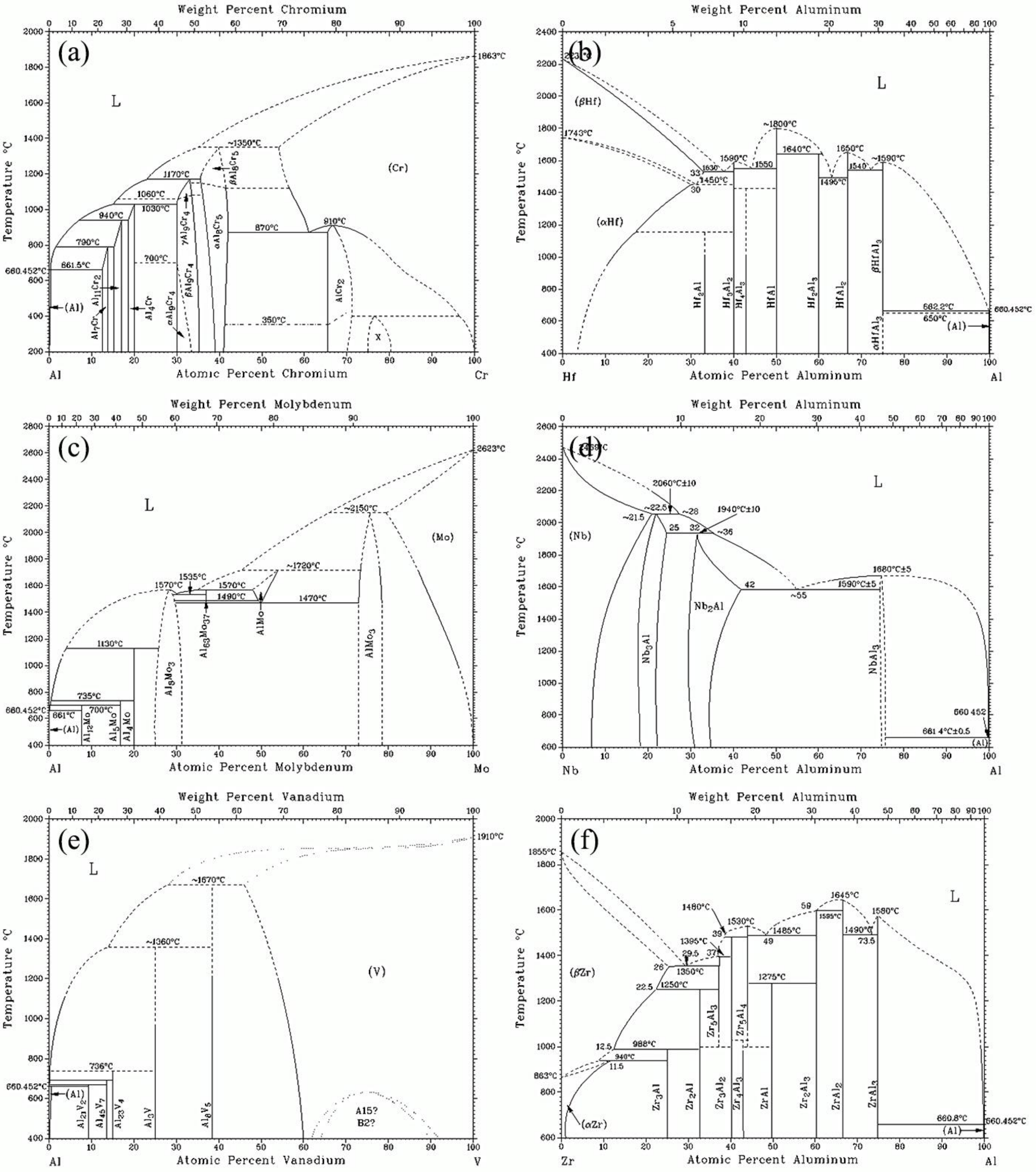

S1. Y-Al phase diagrams where Y is (a) Cr, (b) Hf, (c) Mo, (d) Nb, (e) V, and (f) Zr [18]. Nb and Zr were excluded due to relatively small solubility limit.

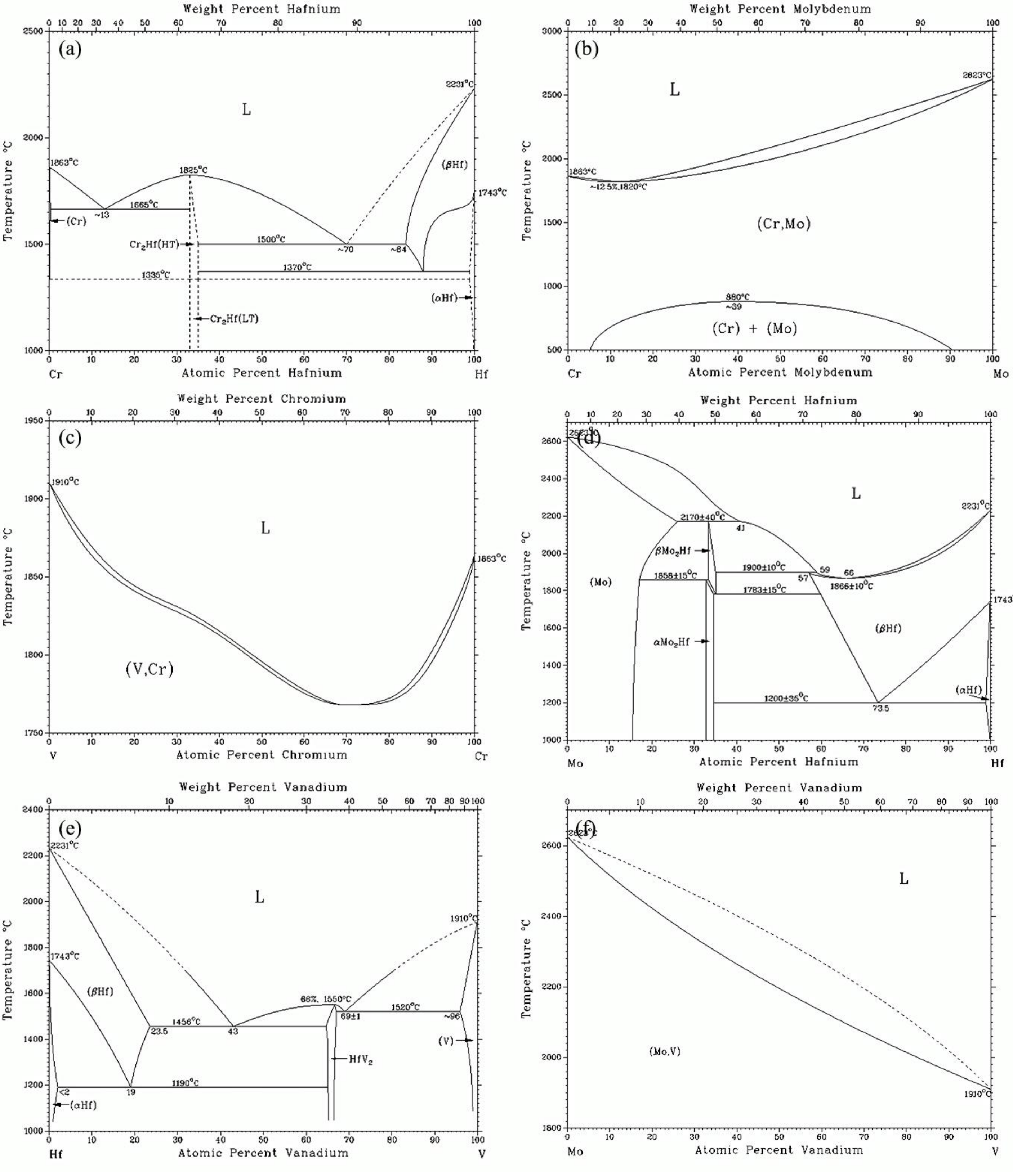

S2. (a) Hf-Cr, (b) Cr-Mo, (c) Cr-V, (d) Hf-Mo, (e) Hf-V, and (f) Mo-V binary phase diagrams [18]. Cr, Mo, and V were selected which have simple phase diagrams.
